# 1512. *Stakeholder Perspectives on Universal HIV Screening in Philadelphia Emergency Rooms*

**DOI:** 10.1093/ofid/ofad500.1347

**Published:** 2023-11-27

**Authors:** Nancy Aitcheson, Caroline O’Brien, Florence Momplaisir

**Affiliations:** Perelman School of Medicine, University of Pennsylvania, Philadelphia, Pennsylvania; Perelman School of Medicine, Philadelphia, Pennsylvania; University of Pennsylvania, Philadelphia, Pennsylvania

## Abstract

**Background:**

Decreasing new HIV infections requires reaching populations at increased risk of acquiring HIV who do not access regular testing. Emergency Departments (EDs) provide a disproportionate amount of care for socially vulnerable populations and are often the only place uninsured patients obtain healthcare. In Philadelphia, where HIV prevalence is 1.2%, further investigation is needed to optimize EDs as HIV testing venues for vulnerable populations. This study sought to elucidate perceived barriers and facilitators to universal HIV screening in EDs among hospital system stakeholders across Philadelphia.

**Methods:**

We conducted a qualitative study using semi-structured interviews with physicians, administrators, and care navigators across five health systems in Philadelphia. Participants were recruited using snowball sampling. An interview guide based on the Consolidated Framework for Implementation Research explored facilitators and barriers to HIV testing in EDs. Interviews were audio-recorded and transcribed verbatim. We analyzed interviews using a modified grounded theory approach to code interview transcripts. Interrater reliability was assessed between multiple coders.

**Results:**

14 individuals were interviewed: 3 registered nurses, 1 social worker, 2 program managers, and 8 physicians. Participants reported EDs play a critical role in providing care to patients who otherwise do not engage with the health care system, who tend to be of lower socioeconomic status and multiply marginalized. The facilitator that most significantly increases ED provider buy-in is a non-ED provider to follow up on HIV test results. Barriers to robust HIV testing programs included competing priorities, lack of personnel, and lack of sustainable funding.

**Conclusion:**

This contextual inquiry revealed shared barriers and facilitators to HIV testing across multiple health systems in a city with a high burden of HIV. The development of a dedicated staff position to deal with indeterminate and reactive HIV testing (removing this responsibility from ED) is the single most important intervention to facilitate HIV testing in EDs. This study provides a clear target for municipal funding in Philadelphia and may be generalizable to HIV testing implementation in EDs in other urban centers.

**Disclosures:**

**All Authors**: No reported disclosures

